# A Billion-Year Facelift and Cleaner Air

**DOI:** 10.1100/tsw.2001.1

**Published:** 2001-01-05

## Abstract

Science leads the first (or is it second?) year of the millennium with a story about a facelift for Jupiter's moon, Ganymede. Nature leads with a story about new regulations on diesel-powered vehicles in the U.S.

*Science* kicks off the New Year with a story about the two faces of Jupiter's giant moon, Ganymede. At last Fall's San Francisco meeting of the American Geophysical Union, planetary scientists reported evidence of a salty, liquid water ocean buried 170 kilometers below the icy crust the moon, reports *Science*. They also discovered clues that may explain why one face of Ganymede is youthful, bright and smooth while the other face is old, dark and pockmarked. Traces of an ancient, now frozen, ocean near the moon's surface could imply that Ganymede was suddenly heated up about one billion years ago and then moved slightly further away from Jupiter. Tidal flexing of the moving moon by Jupiter's gravity could then have stretched out the softened surface, giving one half a shiny new face, reports *Science*. But why did only half the moon get the billion-year facelift? It was just lucky, said the scientists during an interview with *Science*. *Nature* did not cover the story.

The United States could be breathing easier if strict new Environmental Protection Agency (EPA) restrictions on vehicular diesel exhaust have the desired effect, reports *Nature*. A last move by the environment-friendly Clinton administration, the EPA regulations aim to cut emissions of smog-causing nitrogen oxides and airborne particulate matter by requiring diesel trucks and buses to be fitted with pollution control devices similar to catalytic converters. Environmentalists hailed the decision as "the most significant single action to improve US air quality since lead in gasoline was banned in 1986," reports *Nature*. But the results may never be seen if Christine Todd Whitman, the big-oil-friendly Bush administration's selection for director of the EPA, decides to scrap the new rules, says *Nature*. It isn't clear if she will choose to kick off her tenure with such a controversial move Whitman could even turn into an ally of the environmentalists. *Nature* notes that the environmental commissioner from New Jersey during Whitman's tenure as governor once testified in favor of the rule. *Science* did not cover the story.

Both journals also devoted one article to the somewhat surprising decision by the British House of Commons to allow stem cell research in the U.K. The final tally on 19 December was 366 to 174 in favor of changes to the Human Fertilization and Embryology Act of 1990 that, if approved by the House of Lords, will allow such research to take place, reports *Nature*. Given the recent controversy surrounding the use of cells from human embryos, led by anti-abortion groups and the Roman catholic church, scientists were (pleasantly) surprised at the size of the majority voting in favor, reported both journals.
